# Successful treatment with lorlatinib in a patient with meningeal carcinomatosis of *ALK*-positive non-small cell lung cancer resistant to alectinib and brigatinib: A case report

**DOI:** 10.1097/MD.0000000000027385

**Published:** 2021-10-01

**Authors:** Koki Nakashima, Yoshiki Demura, Kosuke Kurokawa, Toshihiro Takeda, Norihiro Jikuya, Masahiro Oi, Toshihiko Tada, Masaya Akai, Tamotsu Ishizuka

**Affiliations:** a Depertment of Respiratory Medicine, Japanese Red Cross Fukui Hospital, 2-4-1, Tsukimi, Fukui-shi, Fukui-ken, Japan; b Third Department of Internal Medicine, Faculty of Medical Sciences, University of Fukui, 23-3 Shimoaizuki, Eiheiji-cho, Matsuoka-gun, Fukui-ken, Japan.

**Keywords:** anaplastic lymphoma kinase, case report, lorlatinib, meningeal carcinomatosis, non-small cell lung cancer

## Abstract

**Rationale:**

Although anaplastic lymphoma kinase (*ALK*) inhibitors are effective treatment options for *ALK*-positive non-small cell lung cancer (NSCLC) with central nervous system (CNS) metastasis, achieving long-term survival in patients with NSCLC with meningeal carcinomatosis resistant to *ALK* inhibitors is difficult. Lorlatinib, a third-generation *ALK* inhibitor, was designed for selective CNS penetration, and exerts potent antitumor activity against tumors resistant to first- and/or second-generation *ALK* inhibitors. However, there is limited information about the activity of lorlatinib in *ALK* inhibitor-resistant meningeal carcinomatosis. Here, we report a case of *ALK*-positive lung adenocarcinoma with meningeal carcinomatosis in which lorlatinib was used after resistance to alectinib and brigatinib.

**Patients concerns:**

A 55-year-old woman with no history of smoking presented to our hospital with a swelling on the left neck. Clinical imaging and histopathological examination revealed a tumor of adenocarcinoma histology in the left upper lung with no CNS metastasis.

**Diagnoses:**

The patient was diagnosed with *ALK*-positive lung adenocarcinoma (cT3N3M1b: stage IVA).

**Interventions:**

She received the second-generation *ALK* inhibitors, alectinib and brigatinib, in the first and second-line settings, respectively. However, she developed meningeal carcinomatosis. Hence, treatment with lorlatinib was initiated in the third-line setting.

**Outcomes:**

The symptoms associated with meningeal carcinomatosis, such as disturbance of consciousness and diplopia, improved dramatically. At 8 months from the initiation of lorlatinib, the patient remained well without disease progression.

**Lessons:**

Lorlatinib is an effective treatment option for patient with *ALK*-positive NSCLC who develop meningeal carcinomatosis resistant to second-generation *ALK* inhibitors. Therefore, lorlatinib should be considered in such cases, even when patients exhibit serious symptoms associated with meningeal carcinomatosis.

## 1 Introduction

Meningeal carcinomatosis is a severe condition associated with poor prognosis in patients with non-small cell lung cancer (NSCLC). Based on previous studies, patients without driver oncogenes have demonstrated a median overall survival of about 1.4 to 5.9 months from diagnosis.^[1,2]^ Contrarily, for patients with NSCLC harboring driver oncogenes who develop meningeal carcinomatosis, molecularly targeted drugs, such as epidermal growth factor receptor (*EGFR*) tyrosine kinase inhibitors (TKIs) and anaplastic lymphoma kinase (*ALK*) inhibitors, have been shown to be effective treatment options.^[1–4]^ However, achieving long-term survival in patients with meningeal carcinomatosis resistant to these drugs is difficult.

Lorlatinib, a third-generation *ALK* inhibitor, was designed for selective central nervous system (CNS) penetration.^[5,6]^ Lorlatinib exerts potent antitumor activity against tumors that are resistant to first- and/or second-generation *ALK* inhibitors.^[7,8]^ Therefore, lorlatinib could be an effective treatment option for patients with *ALK*-positive NSCLC with CNS metastasis, who have been previously treated with *ALK* inhibitors. However, there is limited information about the activity of lorlatinib in *ALK* inhibitor-resistant meningeal carcinomatosis.

We report a patient with *ALK*-positive lung adenocarcinoma who developed meningeal carcinomatosis after treatment with alectinib and brigatinib.

## 2 Case presentation

The patient gave his informed consent for the publication of the details concerning his case, including images.

A 55-year-old woman without a history of smoking presented to our hospital with a swelling in the left neck. Computed tomography revealed a tumor in the left upper lung, swelling of the left supraclavicular lymph nodes, multiple mediastinal lymph nodes, and liver metastasis. Contrast-enhanced magnetic resonance imaging (MRI) of the brain revealed no CNS metastasis. Pathological examination of the endobronchial ultrasound-guided transbronchial needle aspiration of lymph node #7 revealed adenocarcinoma histology. Immunohistochemistry and fluorescence in situ hybridization revealed that the tumor was positive for *ALK*. Based on these findings, a diagnosis of *ALK*-positive lung adenocarcinoma (cT3N3M1b: stage IVA) was establihed.

**Figure 1:**
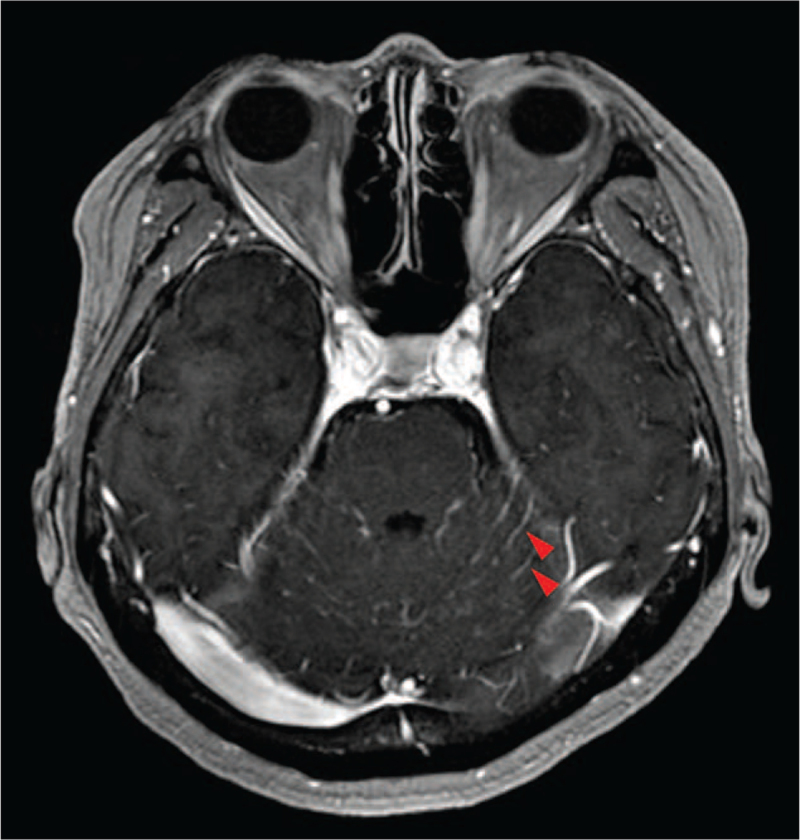
Brain contrast-enhanced magnetic resonance imaging when a disturbance of consciousness and diplopia occurred, shows diffuse, and linear enhancement along the cerebellar folia (arrows).

Alectinib (600 mg/day) was initiated as first-line treatment, and it resulted in a partial response. There were no adverse effects associated with the use of alectinib. However, tumor progression was observed 20 months after the initiation of alectinib. Therefore, brigatinib (180 mg/day) was initiated as second-line treatment, resulting in a partial response. There were no adverse effects associated with the use of brigatinib. However, disturbance of consciousness and diplopia occurred 30 months after the initiation of brigatinib. Contrast-enhanced MRI of the brain revealed a diffuse and linear enhancement along the cerebellar folia (Fig. 1). Examination of the cerebrospinal fluid showed 34 white blood cells /μL (mononuclear cells 95%, polynuclear cells 1%, others 4%), a protein value of 105 mg/dL, and a glucose level of 44 mg/dL. Adenocarcinoma was confirmed by cerebrospinal fluid examination. Based on these findings, a diagnosis of meningeal carcinomatosis was established, and the disturbance of consciousness and diplopia were considered to be its associated symptoms.

**Figure 2:**
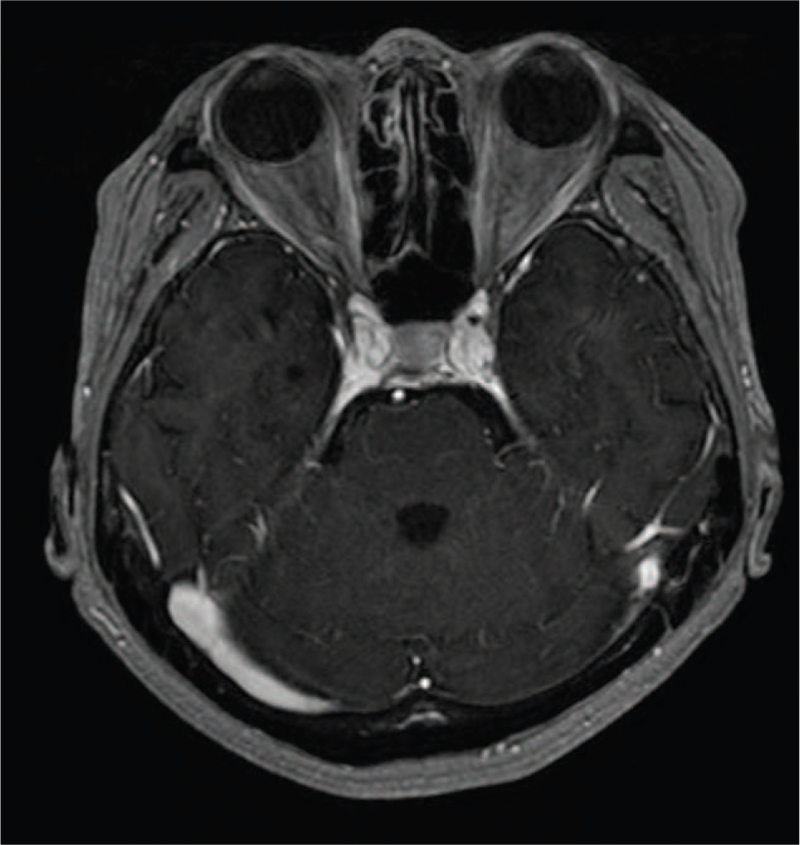
Brain contrast-enhanced magnetic resonance imaging after 3 months after the initiation of lorlatinib shows improvement of the diffuse and linear enhancement along the cerebellar folia.

Treatment with lorlatinib (100 mg/day) was initiated in the third-line setting, and the patient's disturbance of consciousness and diplopia improved dramatically. Contrast-enhanced MRI of the brain revealed that the diffuse and linear enhancement along the cerebellar folia had disappeared (Fig. 2). There were no adverse effects associated with the use of lorlatinib. At 8 months from the initiation of treatment with lorlatinib, the patient remained well without disease progression.

## 3 Discussion

The prognosis of patients with NSCLC who develop meningeal carcinomatosis without driver oncogenes remains poor.^[1,2]^*ALK* inhibitors are an effective treatment option for patient with *ALK*-positive NSCLC who develop meningeal carcinomatosis.^[4]^ However, the successful treatment of *ALK*-positive NSCLC with *ALK*-inhibitor-resistant meningeal carcinomatosis has not yet been reported in the literature. To the best our knowledge, this is the first report of successful treatment with lorlatinib in a patient who responded poorly to alectinib and brigatinib.

The efficacy of lorlatinib in the present case was influenced by 2 factors. First, lorlatinib is highly effective for treating patients with CNS metastasis since it was designed to cross the blood-brain barrier to achieve high CNS exposure.^[5]^ Chen et al^[6]^ have shown that the brain tissue partition coefficient of lorlatinib is 0.7, indicating high CNS exposure. Wang et al^[9]^ reported that lorlatinib was clinically the most effective *ALK* inhibitor against CNS metastasis among lorlatinib, alectinib, brigatinib, and crizotinib. In patients with *EGFR* mutation-positive NSCLC who develop meningeal carcinomatosis, osimertinib has been reported to be the most beneficial treatment option.^[3]^ Several studies have also shown that osimertinib has greater CNS penetration and higher brain exposure than other *EGFR*-TKIs.^[10]^ These results indicate that CNS penetration is an important factor in the treatment of meningeal carcinomatosis with molecularly targeted drugs. In the present case, the high intracranial penetration of lorlatinib likely affected the intracranial metastasis, even though the tumor progressed with brigatinib.

Second, lorlatinib is effective against tumors that are resistant to first-, and second-generation *ALK* inhibitors. Lorlatinib acts against all known *ALK* resistance mutations.^[11,12]^ Several clinical trials have shown that lorlatinib is effective in *ALK*-positive NSCLC patients with secondary *ALK* resistance mutations.^[8,13]^ As seen in the present case, lorlatinib is effective for tumors that are resistant to second-generation *ALK* inhibitors, such as alectinib and brigatinib.

The present report indicates that lorlatinib is an effective treatment option for patient with *ALK*-positive NSCLC who develop meningeal carcinomatosis resistant to second-generation *ALK* inhibitors.

## 4 Conclusion

Here we have reported the successful treatment with lorlatinib of a patient with *ALK*-positive NSCLC who develop meningeal carcinomatosis. Our results suggest that lorlatinib could be considered in patients with meningeal carcinomatosis resistant to second-generation *ALK* inhibitors, even when they exhibit serious symptoms associated with meningeal carcinomatosis.

## Acknowledgments

We would like to thank Editage (www.editage.com) for providing English language editing assistance.

## Author contributions

**Conceptualization:** Koki Nakashima, Yoshiki Demura.

**Data curation:** Yoshiki Demura.

**Resources:** Yoshiki Demura.

**Supervision:** Tamotsu Ishizuka.

**Validation:** Koki Nakashima.

**Visualization:** Koki Nakashima, Yoshiki Demura.

**Writing – original draft:** Koki Nakashima, Yoshiki Demura.

**Writing – review & editing:** Kosuke Kurokawa, Toshihiro Takeda, Norihiro Jikuya, Masahiro Oi, Toshihiko Tada, Masaya Akai, Tamotsu Ishizuka.
